# Diagnostic performance of corneal epithelium- and Bowman’s layer thickness mapping in patients with unilateral Keratoconus

**DOI:** 10.1007/s00417-025-06750-8

**Published:** 2025-01-30

**Authors:** Niklas Pircher, Raphael Kilian, Florian Beer, Ruth Donner, Philipp Roberts, Michael Pircher, Christoph K. Hitzenberger, Julia Aschauer, Gerald Schmidinger, Jan Lammer

**Affiliations:** 1 Department of Ophthalmology and Optometry, Medical University of Vienna, Vienna, Austria; 2 Department of Translational Medicine, University of Ferrara, Ferrara, Italy; 3 Department of Ophthalmology, Ospedali Privati Forli Villa Igea, Forli, Italy; 4 Center for Medical Physics and Biomedical Engineering, Medical University of Vienna, Vienna, Austria; 5 Medical University of Vienna, Waehringer Guertel 18-20, Vienna, 1090 Austria

**Keywords:** Bowman´s layer thickness, Epithelium thickness, Keratoconus, Unilateral ectasia

## Abstract

**Purpose:**

To investigate the role of corneal epithelium- and Bowman’s layer-thickness (ET and BLT) changes as possible early biomarkers of keratoconus (KC) development.

**Methods:**

In this cross-sectional study patients with unilateral KC (UKC) and a group of healthy controls underwent polarization sensitive optical coherence tomography (PS-OCT) for the evaluation of corneal ET and BLT. These values were compared among three subgroups of eyes, i.e., eyes with KC, topographically and tomographically normal fellow eyes (FE) of patients with UKC, and healthy eyes (HE).

**Results:**

Twelve patients with UKC (12 eyes with KC and 12 FE) and 21 healthy age-matched controls (HE) were included. While KC eyes showed the typical epithelial “doughnut pattern” on epithelial maps, there were no differences in ET between FE and HE. BLT was significantly higher in FE compared to HE (average BLT *p* = 0.049, minimum BLT *p* = 0.02, nasal-inferior quadrant *p* = 0.04) as weIl as KC eyes (average BLT *p* = 0.048, minBLT *p* = 0.04, nasal-inferior quadrant *p* = 0.03).

**Conclusions:**

Increase in BLT seems to occur before epithelial thinning in FE of patients with UKC. Since many of KC patients develop the disease bilaterally, Bowman’s layer thickening might represent an early biomarker of KC-development.

## Key messages

*****What is known***:**

- Corneal epithelium thinning and characteristic epithelium thickness patterns such as such the “doughnut pattern” are typical in early keratoconus.

*****What is new ***:**

- Polarization sensitive optical coherence tomography (PS-OCT) has proved to be an effective imaging technique for the evaluation of corneal epithelium- and Bowman’s layer-thicknesses
- Bowman´s layer thickening might take place in the early stages of keratoconus, even before the characteristic epithelial thinning.

## Introduction

Placido-based corneal topography has historically been considered the gold standard imaging-technique for the diagnosis of corneal ectatic diseases [1–3], and even if more recently corneal tomography has allowed for the identification of new characteristic features of early keraconus (KC) such as changes in the posterior corneal elevation [4–6], the diagnosis of subclinical disease remains a challenging task [7]. Several studies have assessed the performance of Scheimpflug-based imaging devices at discriminating healthy and subclinical KC-eyes and showed promising results [8–10]. Available screening indices however, rely mainly on pathological changes in corneal curvatures and do not include features of corneal sublayers [4, 5]. Indeed, visualisation of specific changes in the corneal epithelium or in Bowman’s layer might also be useful in detection of early disease [11–13]. Recent studies using ultrasound imaging [11] and optical coherence tomography (OCT) [14–17], have found that corneal epithelium thickness (ET) alterations such as the so-called “doughnut pattern” are characteristic of almost all early keratoconic eyes [12, 13]. Also, by using a swept source polarization sensitive (PS) - OCT, our group has recently confirmed the presence of pathological changes in the BL of eyes with KC [13]. 

Several publications have described cases of unilateral keratoconus (UKC), where only one eye presents with clinical KC, whereas the fellow eye (FE) shows no topographic signs of corneal ectasia [16, 18]. Since many of the FE will go on developing the disease, we used this model to investigate whether pathological changes of the corneal epithelium and/or BL might precede those of the corneal curvature.

Aim of this trial was to study the changes in corneal epithelium- and Bowman’s layer-thickness in the normal FE of patients with UKC.

## Methods

This cross-sectional study was conducted at the Department of Ophthalmology and the Center for Medical Physics and Biomedical Engineering of the Medical University of Vienna. All investigations adhered to the tenets of the Declaration of Helsinki and the study was prospectively approved by the institutional ethics committee of the Medical University of Vienna (EK Nr: 253/2004). An informed consent was obtained from all included patients.

### Study population

We retrospectively recruited patients with UKC and age-matched healthy controls (healthy right eyes – HE). All participants underwent Scheimpflug imaging (Pentacam HR, Oculus, Wetzlar, Germany) and slit-lamp examination, and the diagnosis of UKC was based on the following findings: (1) topographic characteristics of KC-like central or inferior corneal steepening and asymmetric bow tie with or without skewed axis-astigmatism with a corresponding zone of corneal thinning in one eye; [16, 17] (2) slit-lamp examination features such as Vogt striae, Fleischer ring or apical thinning in one eye; (3) complete absence of any clinical/topographical/pachymetric sign of KC in the fellow eye (FE). Exclusion criteria were previous ocular surgery, corneal cross-linking or trauma and concomitant corneal comorbidities such as corneal scarring. Healthy volunteers with no signs of KC (clinical/topographical/pachymetric) in either eye served as a control group. To avoid corneal warpage, contact lens use was discontinued 4 weeks previous to examination.

### Conical scanning PS-OCT-system

All eyes were studied through a recently introduced custom-designed PS-OCT with conical scanning optics design [19]. The device has already shown its diagnostic potential to distinguish between keratoconic and healthy eyes [13]. Briefly, the system is based on swept-source PS-OCT with a scanning pattern that is generated by a specially designed scanning optics containing an aspheric lens. This allows for an almost perpendicular beam incidence on the corneal surface and results in good signal quality over the entire cornea (limbus-to-limbus). The instrument provides an A-scan rate of 100 kHz and an axial resolution of ~ 6.3 μm. A raster scan pattern was used consisting of 150 B-scans (each containing 1024 A-scans) and was acquired in less than 2 s. During OCT imaging, each participant was positioned in front of the device and was instructed to look at a fixation target. Each eye was scanned 3 times during a single visit. After assessing the quality of the images, the best raster scan volume was chosen for further processing.

### Segmentation of corneal layers and generation of colour-scaled thickness maps

The corneal epithelium- and Bowman’s layer-boundaries were segmented on B-scans using a custom designed software (LabView 2017 SP1, National Instruments). The segmentation algorithm is based on the polarization properties of corneal tissues, and yields highly reproducible thickness maps [20]. Briefly, the PS-OCT system used in this trial incorporates a polarization-sensitive detection unit and records B-scan images of two orthogonal linear polarization states simultaneously. Using image data from these two channels enables good delineation of interfaces such as that of the corneal epithelium and the BL. To compensate for differences in image quality, individual intensity thresholds for detection of every interface in each eye were chosen empirically in one B-scan and were set according to the visually best segmentation results. Apart from setting the thresholds, segmentation was performed fully automatically with the same settings for the entire volume data. Based on the segmentation data lines, en-face thickness maps of corneal layers (i.e., ET and BLT) were computed in an area with a diameter of 11 mm and plotted on a colour scale. For quantitative analysis the maps were subdivided as published recently by our study group [20]. For data evaluation a grid of 25 sectors was chosen, but sectors in the superior and inferior regions (sectors 23–24 and 19–20, respectively) were excluded from quantitative analysis due to obscuring by the upper- and lower eyelid.

### ET and BLT map based parameters

The mean ET and BLT of 21 sectors of the central 11 mm of the cornea was evaluated. The sectors were further grouped into 4 regions: temporal inferior (TI), temporal superior (TS), nasal inferior (NI) and nasal superior (NS). Additionally, the average as well as the thinnest- and the thickest- areas of the corneal epithelium (minET, maxET) and Bowman’s layer (minBLT, maxBLT) were also evaluated.

### Statistical analysis

The ET and BLT of the 4 quadrants as well as the minET, minBLT, the maxET and maxBLT are shown as the mean ± standard deviation (SD). To explore normal distribution, a Shapiro-Wilk analysis was performed. The various thickness values were compared between KC eyes, FE and eyes of healthy subjects, using the ANOVA test with Bonferroni adjustments for multiple comparisons. Post-hoc t-tests were made for differences between each subgroup. The Kruskal-Wallis test was also implemented to confirm ANOVA’s results. A two-sided probability value of ≤ 0.05 was considered statistically significant.

## Results

Twelve patients with UKC (i.e., 12 eyes with manifest KC and 12 normal FE) and 21 healthy age-matched controls (i.e., 21 HE) with a mean age of 32 ± 4 years and 31 ± 7 years, respectively, were included. The mean Kmax of the HE was 44 ± 1 Diopters, whereas that of the KC eyes and FE was 60 ± 1 and 45 ± 2 Diopters, respectively. Analysed data had a normal distribution in each category except for the ETmax in KC eyes, the NI-ET of the healthy group and the BLTmin in HE.

**Fig. 1:**
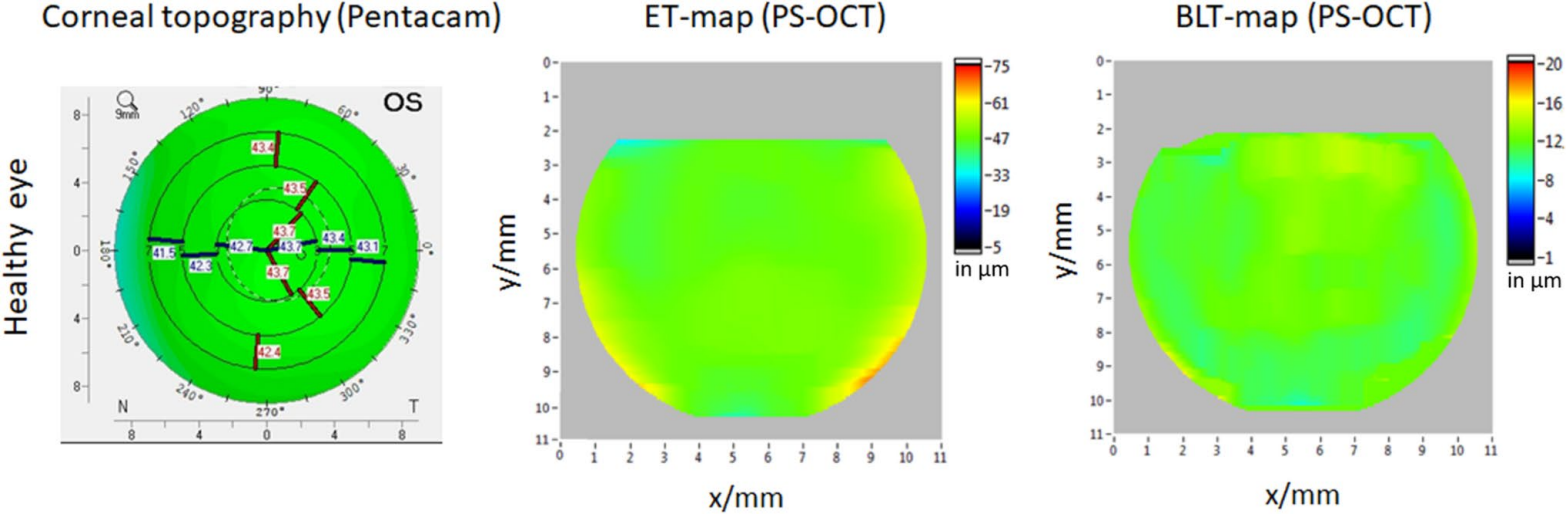
Topography and thickness maps of a representative healthy left eye. Left panel: Corneal topography map recorded by Pentacam HD with no findings suspect for KC. Middle and right panel: ET- and BLT-maps retrieved with PS-OCT. Smooth thickness of the epithelium and the Bowman’s layer can be observed. Color scale bars in diopters (corneal topography) and µm (ET- and BLT-map); ET = epithelial thickness, BLT = Bowman’s layer thickness, PS-OCT = polarization sensitive optical coherence tomography

**Fig. 2:**
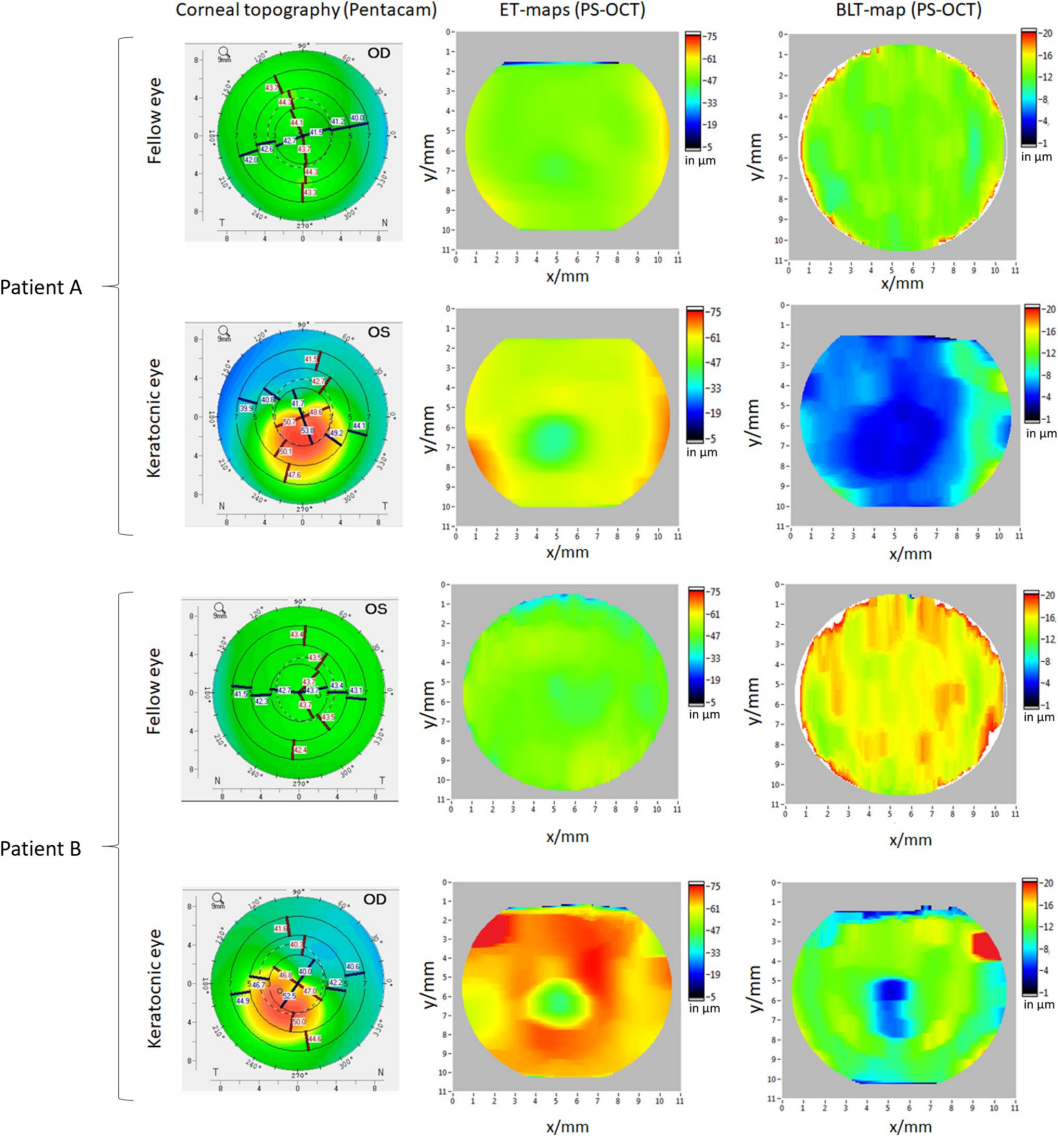
Topography and thickness maps of both eyes of 2 patients with unilateral KC. Top row represents the fellow eye, bottom row represents the KC-eye, respectively Left column: Corneal topography maps recorded by Pentacam HD. These present typical sings of keratoconus in the KC-eyes, but no topographic findings suspect for KC in the fellow eyes Middle column: ET-maps retrieved with PS-OCT. Notice a “doughnut pattern” of epithelial thinning in the area of the corneal apex in the KC-eyes. Fellow eyes show a homogenous thickness of the epithelium Right column: BLT-maps retrieved with PS-OCT. Notice the thinning and irregularity of the BL in the KC-eyes. The BLT en-face maps of the fellow eyes show mild (patient A) to pronounced (patient B) irregular thickening Color scale bars in diopters (corneal topography) and µm (ET- and BLT-maps); ET = epithelial thickness, BL = Bowman’s layer; BLT = Bowman’s layer thickness, PS-OCT = polarization sensitive optical coherence tomography

All eyes in the control group showed homogenous ET- and BLT-maps (Fig. 1), whereas en-face ET and BLT-maps of all KC eyes in the UKC-group showed a “doughnut pattern” at the epithelium level and an irregular thinning of Bowman’s layer (Fig. 2). Accordingly, keratoconic eyes showed a characteristic ET thinning in the area of the KC apex (i.e., minET) and a thicker ET in the surrounding 4 quadrants (epithelial “doughnut pattern”).

Both the ANOVA and Kruskal-Wallis analyses showed significant differences in the TS-ET, NS-ET and in the minBLT between the three subgroups (KC, FE and HE; *p* < 0.05).

T-test results between each group are shown in Table 1. Average BLT was higher in FE than in KC eyes (FE 17 ± 2 μm vs. KC 15 ± 2 μm; *p* = 0.048), as well as minBLT (FE 14 ± 3 μm vs. KC 11 ± 3 μm; *p* = 0.04) and the BLT NI quadrant (FE 17 ± 2 μm vs. KC 14 ± 3 μm; *p* = 0.03).

**Table 1:** Differences in ET and BLT among the different corneal areas between the three subgroups (means and standard deviation) as calculated via t-test. BLT: Bowman’s layer thickness; ET: epithelial thickness; FE: fellow eye; HE: healthy eye; KC: keratoconus; NI: nasal inferior quadrant; NS: nasal superior quadrant; TI: temporal inferior quadrant; TS: temporal superior quadrant; UKC = unilateral keratoconus

Epithelium- and Bowman’s layer-thickness in keratoconic eyes, fellow- and healthy-eyes												
	ET (µm)						BLT (µm)					
	KC	FE	HE	p (KC-FE)	p (KC-HE)	p (FE-HE)	KC	FE	HE	p (KC-FE)	p (KC-HE)	p (FE-HE)
Average	55 ± 5	53 ± 2	54 ± 3	0.21	0.15	0.99	15 ± 2	17 ± 2	15 ± 2	0.048	0.86	0.049
Min	42 ± 6	46 ± 2	46 ± 7	0.08	0.15	0.88	11 ± 3	14 ± 3	12 ± 1	0.04	0.24	0.02
Max	72 ± 3	59 ± 3	61 ± 5	0.12	0.054	0.42	19 ± 2	20 ± 2	20 ± 4	0.33	0.45	0.94
TI	55 ± 1	53 ± 3	52 ± 5	0.48	0.18	0.46	14 ± 3	16 ± 2	14 ± 3	0.06	0.87	0.058
TS	56 ± 3	52 ± 3	52 ± 4	0.008	0.009	0.89	15 ± 3	16 ± 3	14 ± 3	0.41	0.68	0.18
NI	55 ± 8	54 ± 2	52 ± 5	0.56	0.13	0.23	14 ± 3	17 ± 2	14 ± 3	0.03	0.95	0.04
NS	57 ± 4	53 ± 4	50 ± 6	0.057	0.002	0.14	14 ± 2	16 ± 3	15 ± 3	0.12	0.70	0.3

Comparing FE and HE there were no differences in ET between the two groups. Again, average BLT was significantly higher in FE compared to HE (FE 17 ± 2 μm vs. HE 15 ± 2 μm; *p* = 0.049), as well as minBLT (FE 14 ± 3 μm vs. HE 12 ± 1 μm; *p* = 0.02), and the BLT NI quadrant (FE 17 ± 2 μm vs. HE 14 ± 3 μm; *p* = 0.04) and, although not reaching statistical significance, it showed the same trend in the TI sector (FE 16 ± 2 μm vs. HE 14 ± 3 μm; *p* = 0.058).

Interestingly, BLT did not show any statistically significant differences between the KC and HE groups.

## Discussion

Through the years several studies supporting the use of epithelium thickness maps as a diagnostic tool for KC have been published, leaving no doubt that changes in this layer’s thickness are highly characteristic of the disease [12, 16, 21–23]. Furthermore, more than two decades ago, both light and electron microscopy-based studies have demonstrated thinning and breakage of BL in patients with KC [24, 25]. While epithelial remodelling might conceal early KC, modifications in Bowman’s layer seem to occur earlier than corneal stromal changes [24, 25]. 

Although only one eye may be affected initially, KC is typically considered a progressive bilateral disorder [26–28]. Patients diagnosed with unilateral KC (i.e. forme fruste keratoconus in the fellow eye) offer a perfect model to assess early predicting factors for KC, since the normal FE develops KC in up to 20% and 50% of cases respectively at 6 and 16 years from disease onset [26, 29]. If pathological changes of the corneal epithelium or Bowman’s layer precede those in corneal curvature, we would expect to observe them in the fellow topographically normal eye of patients with UKC. These changes can only be evaluated via very high-resolution imaging technologies [13, 30]. In this trial, eyes were analysed with a customized PS-OCT, a device that has already shown to be advantageous over standard imaging modalities when analysing ET and BLT [13]. 

As expected, keratoconic eyes in the UKC-group showed epithelial en-face thickness maps similar to those found in our earlier study [13]. Indeed, a “doughnut pattern” of epithelial thickening around the apex of the cone was found in all en-face maps, most likely representing a highly specific screening parameter for KC [13]. Comparable to our previous paper, eyes with KC also showed an irregular thickness of Bowman’s layer leading to a “moth damage” like pattern on en-face BLT-maps, and fellow eyes had increased BLT compared to both KC and control eyes. However, in this study, the analysis of BLT-values failed to find statistically significant differences between KC- and the HEs, one possible reason for that being the small sample size. As this result appears to be counterintuitive and might have been influenced by a limitation of our study, we suggest further future research should evaluate this important aspect. If our results hold true, this might indicate that BL undergoes a phase of remodeling leading to an increase in its thickness before thinning out again and becoming more irregular once the disease becomes evident. Quantifying the variability or irregularity of BLT might be a more sensitive biomarker to assess changes in the Bowman’s layer integrity.

On the other hand, Bowman’s layer in the FE proved to be significantly thicker in various corneal areas when compared to the KC eyes. This, together with the fact that FE also displayed an overall thicker Bowman’s layer compared to healthy corneas might suggest that during the early developments of KC there actually is a phase of Bowman’s layer remodeling that first leads to a thickening of this layer and only thereafter to it’s thinning, once the disease is evident. A swelling of Bowman’s layer could indicate an incipient disorganisation in this layer’s anatomy and thereby represent an early sign of KC.

In terms of the epithelium, ET values did not significantly differ between FE and HE, suggesting this parameter is not one of the first to change during early phases of the disease. Indeed, FE in the UKC group showed smooth en-face ET-maps comparable to those of the healthy control group.

Li et al. conducted a longitudinal study on patients with UKC and came to the conclusion that approximately 50% of clinically normal fellow eyes will progress to KC within 16 years, with the greatest risk during the first 6 years from the onset [26]. Unfortunately in our study the time of disease onset in patients with UKC was not recorded, therefore we were not able to exactly define the time points at which the analysed anatomical disruptions took place during disease progression.

Among the limitations of this study, its small sample size and the fact that patients were not followed up to detect any disease worsening, represent the most significant ones. Since patients with UKC are rare, complicating the assessment of early predicting factors for KC [27], further investigations and multi-center trials with larger samples are needed to confirm our observations. In fact, a low number of cases reduces the significance of statistical analysis and limits the generalizability of the findings. Furthermore, no biomechanical data were used to classify included eyes. Although (bilateral) asymmetrical keratoconus is a common condition [31], the inclusion of which might have led to disqualify this study’s outcomes, we included only patients with UKC, a rare entity that has been previously described to commonly evolve to a bilateral condition [27]. Finally, although image segmentation was performed fully automatically, the intensity thresholds for interface detection were set manually, which although made meticulously, might represent a source of error.

This study shows that Bowman’s layer in FE of patients with UKC is significantly thicker compared to that of normal- and KC-eyes suggesting that BLT irregularities and swelling as seen on PS-OCT might serve as a marker for the diagnosis of pre-clinical KC. Given the rarity of this condition, we encourage further multicentric longitudinal research to validate this study’s results. These studies should have large sample sizes and ideally include some histological findings as well.
